# Fusion gene heterogeneity and kinase enrichment in high-grade serous carcinomas

**DOI:** 10.1016/j.neo.2026.101332

**Published:** 2026-06-25

**Authors:** Ioannis Panagopoulos, Ane Stranger, Anita Sveen, Mie Jareid, Susanne G. Kidd, Katharina Bischof, Kjetil Taskén, Anne Dørum, Ben Davidson, Ragnhild A. Lothe, Rolf I. Skotheim, Bjarne Johannessen

**Affiliations:** a Department of Molecular Oncology, Institute for Cancer Research, Oslo University Hospital-Radiumhospitalet, Oslo, Norway; b Institute of Clinical Medicine, Faculty of Medicine, University of Oslo, Oslo, Norway; c Department of Surgical Oncology, Section of Gynaecological Oncology, Oslo University Hospital-Radiumhospitalet, Oslo, Norway; d Department of Cancer Immunology, Institute for Cancer Research, Oslo University Hospital-Radiumhospitalet, Oslo, Norway; e Department of Pathology, Oslo University Hospital-Radiumhospitalet, Oslo, Norway; f Department of Informatics, Faculty of Mathematics and Natural Sciences, University of Oslo, Oslo, Norway

**Keywords:** High-grade serous tubo-ovarian carcinoma, Fusion gene heterogeneity, RNA sequencing, Kinase enrichment, Homologous recombination deficiency

## Abstract

Genomic instability in high-grade serous tubo-ovarian carcinoma (HGSTOC) can generate fusion genes with potential roles in tumor evolution and clinical relevance. HGSTOCs commonly disseminate prior to diagnosis, but most studies analyze only one tumor sample per patient, limiting understanding of fusion gene dynamics. Here, RNA sequencing was performed on 108 tumor samples from multiple intra‑abdominal sites in 23 patients. A consensus approach involving three fusion callers identified 170 high-confidence fusion genes present in all samples from each patient, most of which were non‑recurrent and not in-frame.

Protein kinases were significantly enriched among fusion partners, and commonly retained intact catalytic domains. Several of the kinase fusion genes may have oncogenic relevance, including *AKAP8L::BRD4* (recurrent in three patients), *GPBP1L1::MKNK1, SPAG16::ERBB4, UGP2::MAPK4*, and *STRN3::PRKD1*. Four fusions involved homologous recombination-related genes, including *FUT10::SPIDR, RAD54B::VIRMA, RAD54L::PLXNA2*, and *SMARCAD1::BOD1L1*. Integration of these fusion genes with other somatic alterations in homologous recombination-related genes, deficiency signatures, and germline *BRCA1*/*2* variants increased the involvement of the homologous recombination pathway to 15 HGSTOCs (65%). Overall, HGSTOC has a heterogeneous fusion gene landscape, and multi-site sampling with consensus calling offers a robust strategy for resolving fusion profiles.

## Highlights

• Multi-site RNA sequencing reveals fusion gene heterogeneity in tubo-ovarian cancer.
• Most fusion genes are nonrecurrent and unique to individual cancers.
• Protein kinases are significantly enriched among fusion partners.
• Several kinase fusions retain intact catalytic domains.
• Fusion genes and HRD alterations broaden homologous recombination involvement.

## Introduction

High-grade serous tubo-ovarian carcinoma (HGSTOC) accounts for approximately 80% of ovarian cancer related deaths [1]. The hallmark molecular feature of HGSTOC is the somatic mutation of *TP53*, accompanied by extensive genomic aberrations [2]. Approximately 20% of HGSTOCs also exhibit *BRCA1/2* inactivation due to germline or somatic mutations, or epigenetic alterations. An additional 30% of HGSTOCs have other forms of homologous recombination deficiency (HRD) [2]. These patients may benefit from maintenance therapy with poly (ADP-ribose) polymerase inhibitors, especially if given after first-line chemotherapy and in cases of platinum sensitivity [3].

Fusion genes are somatic structural variants generated by DNA breakage and aberrant repair that join two separate transcriptional units (fusion partner genes) into a single hybrid gene. These events can disrupt normal gene regulation or lead to the production of chimeric proteins with oncogenic potential [4]. Owing to their cancer-specific nature, fusion genes are attractive as diagnostic and prognostic biomarkers, therapeutic targets, and sources of neoantigens for immunotherapy [5]. High-throughput RNA sequencing enables the detection of expressed fusion transcripts derived from such genomic rearrangements [6]. Fusion transcripts represent individual chimeric RNA isoforms and may differ in exon composition as a result of alternative splicing, including variable inclusion or exclusion of exons from either fusion partner. As a consequence, multiple distinct fusion transcripts can arise from the same underlying genomic rearrangement. These transcript variants therefore represent a single fusion gene event, defined by the involved gene pair (e.g., multiple *Gene1::Gene2* fusion transcripts corresponding to one *Gene1::Gene2* fusion gene).

In HGSTOC, extensive genomic rearrangements, together with the absence of recurrent oncogenic driver alterations beyond ubiquitous *TP53* mutations, have led researchers to propose gene fusions as potential contributors to tumorigenesis [7,8].

Numerous studies have identified fusion events in HGSTOC, but available data suggest that their recurrence across patients is relatively low [7,8]. Furthermore, intra-patient heterogeneity of fusion events across primary and metastatic tumor sites might impact the biomarker and/or therapeutic potential, but this remains poorly understood.

This study examines fusion events (1) in primary and metastatic tumor sites from the same patient, to identify fusions that emerge early in HGSTOC evolution and persist through metastasis, and (2) across patients, to identify potential recurrent fusions.

## Materials and methods

### Sex as a biological variable

This study investigated cancer of the female reproductive system.

### Patients and sample collection

The present study included 23 patients with disseminated HGSTOC who underwent either primary (17 patients) or interval (6 patients) cytoreductive surgery following platinum-taxane chemotherapy at Oslo University Hospital-*Radiumhospitalet*, between 2002 and 2012. Patient selection was based on diagnosis, consent, and the availability of fresh-frozen tissue samples suitable for genomic and transcriptomic analysis, emphasizing both intra- and inter-tumor heterogeneity through double sampling of multiple tumor sites. Clinical information such as patient age at surgery, tumor characteristics (topography, histology, grade and stage), chemotherapy details (type, timing and response), and survival times (progression-free and overall) were described elsewhere [9].

A gynecologic pathologist (B.D.) reviewed hematoxylin and eosin-stained sections of the fresh-frozen samples to confirm the diagnosis and estimate tumor purity. For samples collected prior to 2014, the ovary was designated as primary site; the site of origin was previously assessed [9]. From each patient, a total of 3-7 samples from ovary and 1-2 samples from extraovarian sites were collected, resulting in a total of 108 samples from 9 different anatomical sites: 58 ovarian, 47 solid extraovarian, and 4 ascites [9]. The tumor samples were assessed to have a median cancer cell content of 70% (10-90th percentile range 40%−90%). One sample was later excluded due to low tumor purity; therefore, the samples are numbered 1-109 with #79 missing. For patient ROC2-1730, the ovarian (samples 87-89) and extraovarian (samples 90-93) cancers harbored different *TP53* mutations as well as mutations affecting different genes, supporting a polyclonal cancer origin [9]. Thus, samples 87-89 were designated cancer A, whereas samples 90-93 were designated cancer B. All samples are registered in the Biobank Registry of Norway (S-04300).

### RNA extraction, transcriptome sequencing, and sequence data processing

Total RNA was isolated using the AllPrep DNA/RNA/miRNA Universal kit (Qiagen GmBH, Hilden, Germany). Whole transcriptome sequencing was performed by the Genomics Core Facility at the Institute for Cancer Research, The Norwegian Radium Hospital, Oslo University Hospital. RNA sequencing libraries were prepared from 1 µg of total RNA using TruSeq Stranded Total RNA Library Prep Kit with Ribo-Zero Gold (Illumina Inc., San Diego, CA, USA). Paired-end sequencing (2 × 101 base pairs) was conducted on a NovaSeq6000 sequencing platform (Illumina). The mean number of uniquely mapped RNA sequence reads was 70,359,353 per sample.

RNA sequencing reads were trimmed and assembled using TRIMMOMATIC v.0.38 and stored as fastq files [10]. Tumor purity was estimated using Estimate v1.0.13, a method that uses gene expression signatures to infer the fraction of stromal and immune cells in tumor samples [11].

### Fusion transcript prediction, functional annotation and enrichment analyses

To predict fusion transcripts, we used STAR-Fusion v1.13.0 [12], Arriba v2.4.0 [13] and FusionCatcher v1.30 [14], with the human genome library GRCh38 (GRCh38_gencode_v33_CTAT_lib_Apr062020) and fastq files as input. Default parameters were applied. Genomic localization of the fusion breakpoint and coding sequence was examined using the UCSC Genome Browser [15] and BLAT tool [16]. Putative driver fusions or biomarkers that overcome tumor heterogeneity were defined as fusion events consistently detected across all tumor samples from a given patient. Fusion genes were included in the analysis if they met at least one of the inclusion criteria: First, under a strict consensus rule, the same fusion transcript with identical breakpoints had to be detected across all tumor samples from the same patient and by all three fusion detection tools (STAR-Fusion, Arriba, and FusionCatcher). Second, under an expanded consensus rule, the same fusion gene (*Gene1::Gene2*) had to be detected across all tumor samples from a given patient, even if the exact fusion transcript breakpoints differed across samples or algorithms, or if one fusion caller did not detect the fusion gene in every sample, provided that the event was supported by at least two of the three tools. This approach ensured that biologically relevant fusion events were retained despite transcript variation or tool-specific sensitivity differences.

In addition to the NCBI database [17] (https://www.ncbi.nlm.nih.gov/), specialized databases were searched to annotate the fusion partner genes for relevant functional implications, such as kinases, transcription factors, proteins with signal peptides, and DNA repair proteins (see below in statistical analysis).

To obtain gene-level expression values, RNA-seq reads were quantified using the HTSeq tool (version 2.0.2), which counts the number of sequencing reads overlapping each annotated gene, producing raw gene-level count data [18]. Protein-coding and lncRNA genes with >10 raw HTSeq counts in at least three of the 108 samples were retained, resulting in 26,113 genes for downstream analysis. Normalization and variance-stabilizing transformation were performed using the DESeq2 statistical package (version 1.38.3), which is designed for modelling RNA-seq count data and correcting for differences in sequencing depth [19]. The vst() function was applied to obtain variance-stabilized, library-size-normalized expression values.

### Assessment of HR pathway involvement

To determine the involvement of HR in each cancer, we integrated previously published HRD-signature status and known pathogenic germline *BRCA1/2* variants [9] with genomic findings derived from exome sequencing and fusion analysis. Genes involved in the HR pathway were defined according to the Kyoto Encyclopedia of Genes and Genomes (KEGG), using the KEGG BRITE database (Functional Hierarchies of Biological Entities, updated October 2025) [20]. Whole-exome sequencing and mutation-calling methodologies were described previously [9]. Nonsynonymous exonic single nucleotide variants (SNVs), frameshift indels, and splice-site mutations (SNVs or indels) were considered nonsilent, and only variants detected in all samples from the same patient (or in all samples of cancer A and all samples of cancer B of patient ROC2-1730) were included in the assessment. Based on this integrated approach, cancers were classified into two categories: (1) Involvement, defined as the presence of an HRD-signature or a pathogenic germline variant or a somatic alteration affecting an HR gene, including nonsilent variants and fusion genes involving HR genes; and (2) No detected involvement, defined as the absence of HRD-signatures and the absence of HR-related genomic alterations.

### Statistics

To evaluate differences in the number of fusion transcripts identified by the three algorithms across the 108 matched samples, we applied the non-parametric Friedman test for repeated measures. When the overall test was significant, post-hoc pairwise comparisons were carried out using the Wilcoxon signed-rank test with Bonferroni correction (adjusted significance level α = 0.0167). The over-representation of selected functional categories, including kinases, transcription factors, SignalP-predicted secreted proteins, DNA repair genes, and non-coding RNAs, among fusion-partner genes was assessed using the hypergeometric test [21]. The background population (N) was based on the GENCODE 2025 report [22] and defined as approximately 19,500 human protein-coding genes. For analyses involving non-coding RNAs, this number was extended to ∼55,500 genes, including both protein-coding and 36,000 annotated non-coding RNA genes. Pseudogenes and other non-transcribed loci were excluded. For each category, the number of genes in the population belonging to that category (K) was determined from established annotations (see below). Statistical calculations were based on the number of observed genes from each category among the 322 unique fusion-partner genes identified across 170 fusion genes, after collapsing duplicates and reciprocal events. Enrichment was assessed using the one-sided hypergeometric test. P-values were adjusted for multiple testing using the Benjamini–Hochberg (BH) procedure [23]. All statistical analyses were performed in Python 3.12.7 using the SciPy 1.13.1 statistics module (scipy.stats.hypergeom, scipy.stats.friedmanchisquare, scipy.stats.wilcoxon) within the Anaconda distribution [24].

Protein kinases were defined according to the curated human kinome from KinHub (http://kinhub.org/kinases.html; accessed November 12, 2025) and the original classification by Manning et al. [25]. Together, these resources comprise 536 genes encoding protein kinase-like (PKL) domains, including typical, atypical, and pseudokinases (e.g., *SCYL1* and *BRD4*). Because some definitions of the human kinome also include lipid kinases, we performed a complementary analysis in which the 19 phosphoinositide kinase genes were added to the PKL set [26]. The kinase population was therefore defined either as K = 536 (PKL only) or K₁ = 555 (PKL + lipid kinases), and enrichment was computed for both definitions using the same hypergeometric framework. The PI3K-related protein kinase MTOR was already included in the PKL set (20 genes coding for protein kinase), whereas *PIK3CA* was included only in the expanded analysis (21 genes). Transcription factors were defined according to the Transcription Factor Checkpoint 2.0 [27]. For this category, the population was defined as K = 3,554, corresponding to all human genes annotated as transcription factors in this database. Among the fusion-partner genes detected in our dataset, n = 57 were transcription factors. Genes encoding proteins with a signal peptide were defined according to the UniProtKB/Swiss-Prot annotations reported by Gutierrez-Guarnizo et al. [28]. In that study, the authors identified 3,607 human genes encoding proteins with annotated signal peptides, and this number was therefore used as the background population for the signal peptide category (K = 3,607). Among the 322 unique fusion-partner genes in our dataset, n = 28 were annotated as encoding proteins with a signal peptide. DNA repair genes were defined according to the Kyoto Encyclopedia of Genes and Genomes (KEGG), using the KEGG BRITE database (Functional Hierarchies of Biological Entities, updated October 2025) [20]. The analysis was based on the hierarchical classification hsa03400: DNA Repair and Recombination Proteins, which groups genes into major repair pathways, including base excision repair (BER), nucleotide excision repair (NER), mismatch repair (MMR), HR, non-homologous end-joining (NHEJ), and the Fanconi anemia (FA) pathway. The background population was defined as K = 356, corresponding to the unique set of human DNA repair genes extracted from these six KEGG pathways after removal of duplicates. Among the 322 fusion-partner genes in our dataset, n = 8 were annotated as DNA repair genes. Non-coding RNA genes were defined according to GENCODE v47 [22], which provides a comprehensive reference annotation of the human genome and includes both long non-coding RNAs (lncRNAs) and small non-coding RNAs, such as microRNAs (miRNAs), small nucleolar RNAs (snoRNAs), and small nuclear RNAs (snRNAs). For this category, the background population was defined as K = 36,000, corresponding to the total number of non-coding RNA genes reported in the GENCODE v47 release. This annotation includes approximately 56,000 transcripts in total, spanning both coding and non-coding RNA species. Among the 322 unique fusion-partner genes in our dataset, n = 34 were annotated as non-coding RNA genes. To evaluate whether expression of the ten kinase genes with fully retained catalytic domains in their detected fusion transcripts differed between cancers, we used a non-parametric statistical approach. This statistical approach was motivated by the small and unequal number of samples per cancer (ranging from 3 to 7) and because the group-wise expression distributions could not be assumed to follow a normal distribution. For each kinase, global differences in expression across the 24 cancers were first assessed using the Kruskal-Wallis test [29]. When the overall test was significant, pairwise post-hoc comparisons were performed using Dunn’s test [30] with Holm’s sequential correction for multiple testing [31]. After establishing statistically significant differences, variance-stabilized expression values were summarized using quartile-based categories to facilitate cancer-level interpretation. For each kinase, cancers were classified as high-expression, medium-expression, or low-expression based on whether their expression values fell within the upper 25%, middle 50%, or lower 25% of the distribution, respectively. These quartile-derived categories were subsequently integrated with the fusion dataset to determine whether cancers harboring domain-intact kinase fusions exhibited correspondingly altered expression patterns. This two-step strategy allowed us to distinguish between cancers that simply ranked high or low and those that were statistically different from others in a non-parametric framework.

## Results

### Consensus detection of ubiquitously expressed fusion genes across tumor sites

**Fig. 1:**
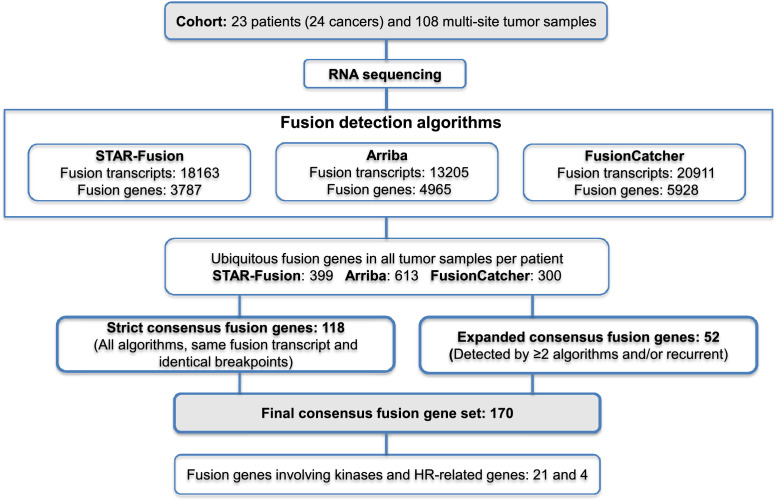
Overview of the fusion gene detection, filtering, and consensus strategy. Schematic overview of the analytical workflow used for fusion gene identification and prioritization. RNA sequencing was performed on 108 multi-site tumor samples obtained from 23 patients, including 24 cancers due to one patient with two synchronous cancers. Fusion transcripts were independently detected using three algorithms: STAR-Fusion, Arriba, and FusionCatcher, yielding large initial sets of candidate fusion transcripts and fusion genes for each method. For each algorithm, fusion genes that were consistently detected across all tumor samples from the same patient were retained as ubiquitously expressed fusion events. To derive a high-confidence fusion gene set, results were integrated across algorithms using two consensus levels: a strict consensus, requiring detection by all three algorithms with identical fusion transcripts and breakpoint coordinates, and an expanded consensus, including fusion genes supported by at least two algorithms and/or recurrent across patients. Application of these criteria resulted in a final curated set of 170 consensus fusion genes, which was used for all downstream analyses. From this set, fusion genes involving kinases and homologous recombination (HR)–related genes were further examined in dedicated analyses.

Total RNA sequencing was performed on 108 multi-site tumor samples from 23 patients, followed by independent detection of fusion transcripts using three algorithms: STAR-Fusion, Arriba, and FusionCatcher. Transcript variants arising from the same gene-pair rearrangement were consolidated into a single fusion event, and only events identified in all samples from each patient were retained in the final consensus set. An overview of the fusion detection and filtering strategy is shown in Fig. 1.

**Fig. 2:**
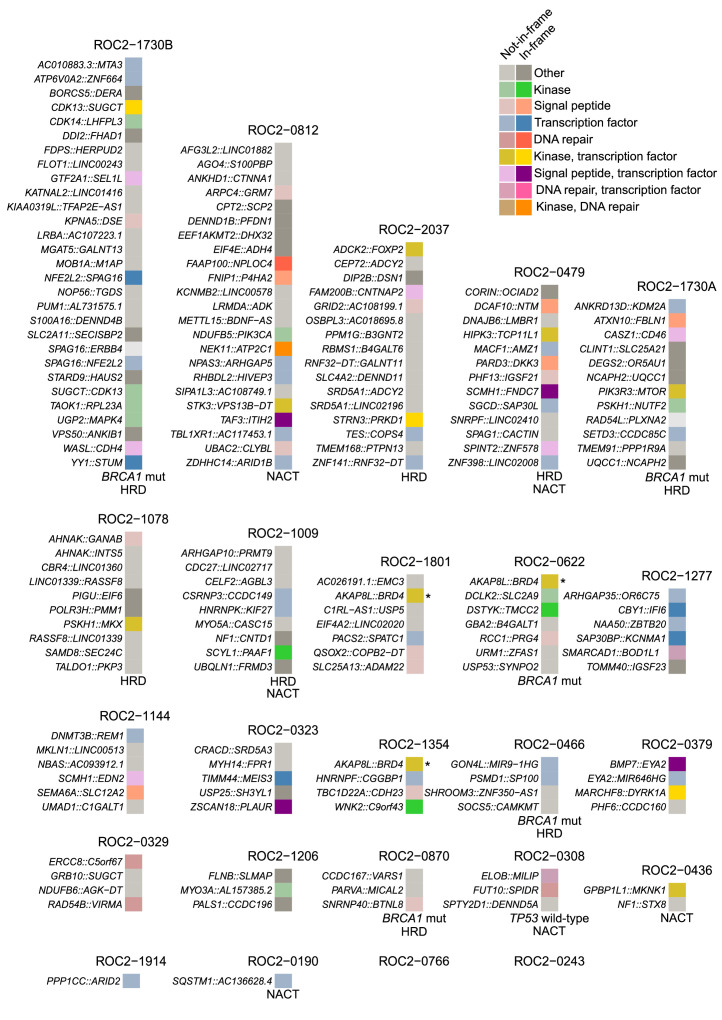
Fusion genes detected in high-grade serous tubo-ovarian carcinomas (HGSTOC). The figure summarizes the 170 fusion genes concurrently detected by STAR-Fusion, Arriba, and FusionCatcher. The *AKAP8L::BRD4* fusion (*) was identified by all three algorithms in multiple patient samples. Fusion transcripts, both in-frame and not-in-frame, are color-coded according to whether the 5′ or 3′ partner gene encodes a kinase, signal peptide–containing protein, transcription factor, or DNA repair protein. Not-in-frame is a collective category that includes out-of-frame fusions, transcripts with premature stop codons, fusions involving untranslated regions, and chimeras incorporating non-coding elements such as microRNAs, antisense RNAs, long non-coding RNAs, long intergenic non-protein-coding RNAs, or divergent transcripts. Two patients (ROC2-0766 and ROC2-0243) did not harbor fusion genes consistently detected across all examined samples. Additional patient-level annotations, i.e., pathogenic germline *BRCA1/2* variants, homologous recombination deficiency (HRD) detected by HR signatures, neoadjuvant chemotherapy (NACT) treatment, and wild-type TP53 status, are shown.

The three algorithms differed substantially in the total number of fusion transcripts and fusion events detected across the dataset (Friedman test, χ² = 116.26, p = 5.68 × 10⁻²⁶; Fig. 1; Supplementary Tables S1 and S2). The fusion detection rates also differed in all pairwise algorithm comparisons (Arriba vs. STAR-Fusion: p = 1.71 × 10⁻¹⁵; Arriba vs. FusionCatcher: p = 1.38 × 10⁻¹⁷; STAR-Fusion vs. FusionCatcher: p = 1.38 × 10⁻⁶; Wilcoxon signed-rank tests with Bonferroni correction), supporting the use of a multi-algorithm consensus strategy for robust fusion detection. Furthermore, only a subset of detected fusion events was consistently identified across all tumor samples from the same patient (STAR-Fusion: 10.5%; Arriba: 12.3%; FusionCatcher: 5.1%; Fig. 1). A consensus approach that combined cross-sample consistency with multi-algorithm support yielded a final set of 170 high-confidence fusion genes, which was used for all downstream analyses (Fig. 2, Supplementary Tables S3-S5).

**Fig. 3:**
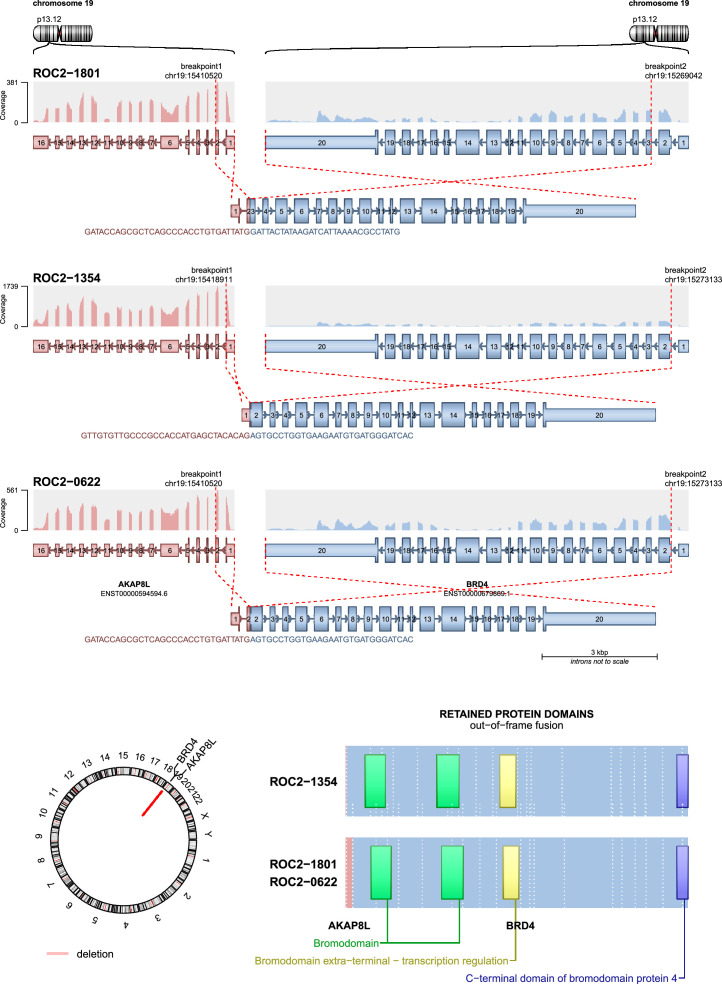
Recurrent *AKAP8L::BRD4* fusion detected by three fusion-calling algorithms in high-grade serous tubo-ovarian carcinomas. The *AKAP8L::BRD4* fusion was identified independently by STAR-Fusion, Arriba, and FusionCatcher in tumors from three patients (ROC2-1354, ROC2-1801, and ROC2-0622). The illustration, generated with Arriba, displays exon structures, read coverage, and breakpoint coordinates for the 5′ partner *AKAP8L* and the 3′ partner *BRD4*. The lower panel summarizes the protein domains retained in the chimeric transcripts.

Consensus fusion genes were detected in 21 of the 23 patients, including two synchronous HGSTOCs from patient ROC2-1730 (designated ROC2-1730A and ROC2-1730B), resulting in a detection frequency of 92% (22 of 24 cancers). However, all fusions except one were unique to a single patient (Fig. 2, Tables S3-S5). The *AKAP8L::BRD4* fusion was detected by all three algorithms in three patients (ROC2-1354: all four samples, ROC2-1801: six of seven samples, ROC2-0622: one of three samples). The corresponding fusion events (genes and transcripts) and breakpoint details are illustrated in Fig. 3 and Supplementary Tables S3-S5.

There was considerable variation in the number of consensus fusion genes across patients, ranging from 0 to 29 (Fig. 2). Four patients had none (ROC2-0243 and ROC2-0766) or only one common fusion gene across samples (ROC2-0190 and ROC2-1914), while others had a much higher number (ROC2-1730B with 29 fusion genes, ROC2-0812 with 23, ROC2-2037 with 16, and ROC2-0479 with 13) (Fig. 2 and Supplementary Tables S3-S5).

Fusion genes arose from both interchromosomal and, more frequently, intrachromosomal rearrangements (Supplementary Tables S3-S5). Reciprocal fusion events with consistent underlying chromosomal aberrations provided additional support for several structural aberrations, including *LINC01339::RASSF8* and its reciprocal *RASSF8::LINC01339* in patient ROC2-1078, and *NCAPH2::UQCC1* together with *UQCC1::NCAPH2* in ROC2-1730A (Supplementary Tables S3-S5). A complete list of reciprocal fusions identified by Arriba is provided in Supplementary Table S6.

The majority of fusion transcripts were not-in-frame and included out-of-frame fusions, transcripts with premature stop codons, fusions involving untranslated regions, and fusions incorporating non-coding elements such as microRNAs, antisense RNAs, long non-coding RNAs, long intergenic non-protein-coding RNAs, and divergent transcript (Fig. 2, Supplementary Tables S3-S5).

### Over-representation of kinases among fusion gene partners

Most fusion partners were protein-coding genes, whereas only 34 (10.6%) were annotated as non-coding RNAs. To assess whether predefined functional gene categories were overrepresented among fusion-partner genes, we examined the distribution of the 322 unique genes involved as fusion partners in the 170 fusion events across multiple biologically relevant functional classes, as defined in the Materials and Methods. Among these categories, only kinase genes showed significant enrichment among fusion-partner genes (Supplementary Table S7). Protein kinases were over-represented 2.26-fold (20 observed vs. 8.85 expected; FDR = 0.0022), and the combined kinase category (protein and phosphatidylinositol kinases) showed a similar 2.29-fold over-representation (21 observed vs. 9.16 expected; FDR = 0.0022). Signal peptide-containing genes and non-coding RNAs appeared to be under-represented relative to their background frequencies (Supplementary Table S7).

In total, 11 patients had cancers harboring kinase fusions, and in patient ROC2-1730, kinase rearrangements were present in both synchronous cancers (Supplementary Table S8). Some cancers harbored a single kinase fusion, whereas others harbored two or more distinct kinase fusions. Analysis of domain architecture across the 21 unique kinase fusion events (Supplementary Table S8) showed that 10 of these fusions (47.6%) retained a fully intact kinase domain, preserving 100% of the catalytic sequence irrespective of reading-frame status (Supplementary Fig.s S1-S9; Supplementary Tables S3-S5 and S8).

### Expression of fusion-involved kinase genes with fully retained kinase domains

To evaluate whether tumor samples differed significantly in the expression of fusion-involved kinases with retained kinase domains, we performed Kruskal-Wallis tests for each of the ten genes included in the analysis (*PRKD1, MAPK4, MKNK1, DYRK1A, BRD4, PIK3CA, SCYL1, MTOR, CDK13*, and *ERBB4*; Supplementary Tables S9-S10). Each tumor sample was treated as an independent observation, reflecting potential spatial heterogeneity between sampled sites. All genes showed significant global differences in expression (unadjusted p-values ranging from 1.36 × 10⁻⁶ to 5.58 × 10⁻¹⁰), and all remained significant after Holm’s correction across genes.

**Fig. 4:**
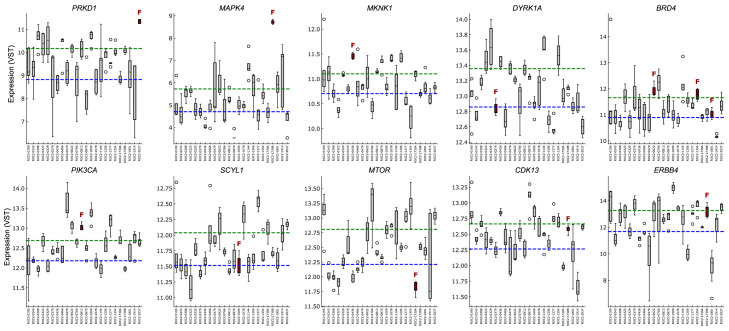
Expression profiles of ten kinase genes with fully retained catalytic domains in their fusion transcripts, illustrating intra- and inter-cancer variability. Each panel shows VST-normalized expression values for one kinase across all cancer samples. Boxplots summarize the distribution of sample-level expression values within each cancer (ROC2-1730A and ROC2-1730B shown separately), with the horizontal line indicating the median and the box representing the interquartile range. Cancers harboring a fusion involving the corresponding kinase gene are highlighted by dark red boxplots and annotated with a bold “F”. Horizontal dashed lines indicate the 25th (blue) and 75th (green) percentiles of patient-level mean expression values across all cancers for each kinase. VST, variance-stabilizing transformation.

To determine which samples contributed to these differences, we performed pairwise post-hoc comparisons using Dunn’s test with Holm correction for multiple testing (Supplementary Table S11). This analysis showed that numerous sample pairs differed significantly, indicating that the global heterogeneity was driven by subsets of samples with particularly high or low expression levels. The specific relationship between expression and fusion status was evaluated separately at the cancer level (Fig. 4).

To examine this association, we summarized kinase expression per cancer using patient-level mean VST-normalized values and applied quartile-based thresholds, defining cancers in the top 25% as high-expression and those in the bottom 25% as low-expression for each kinase (Fig. 4; Supplementary Table S12).

Integration of these expression categories with fusion status revealed clear patterns linking expression level to the presence of domain-intact kinase fusions (Supplementary Table S13). Several fusion-positive cancers fell into the high-expression group for their respective kinases, including ROC2-2037 (*STRN3::PRKD1*), ROC2-1730B (*UGP2::MAPK4*), ROC2-0436 (*GPBP1L1::MKNK1*), ROC2-0622 and ROC2-1354 (*AKAP8L::BRD4*), and ROC2-0812 (*NDUFB5::PIK3CA*), indicating that these rearrangements are often observed in cancers with elevated expression of the affected kinase gene. Conversely, other fusion-positive cancers showed low expression of the same kinase, including ROC2-0379 (*MARCHF8::DYRK1A*), ROC2-1009 (*SCYL1::PAAF1*), and ROC2-1730A (*PIK3R3::MTOR*), suggesting that not all domain-intact kinase fusions are associated with overexpression and that regulatory context may differ markedly across cancers. Overall, kinase fusion genes with retained catalytic domains displayed cancer-specific expression patterns, ranging from marked overexpression in some cancers to pronounced down-regulation in others (Fig. 4; Supplementary Table S13).

### Evaluation of HR pathway involvement

HR involvement was evaluated by integrating HR deficiency (HRD) signature status, pathogenic germline *BRCA1/2* variants, and somatic alterations, including nonsilent variants and fusion genes, in KEGG-annotated HR genes (see Methods; Supplementary Table S14). Based on this combined assessment, 15 of the 24 cancers (62.5%) showed involvement of the HR pathway (Table 1). This group comprised all cancers with HRD-signatures, five cancers carrying pathogenic germline *BRCA1/2* variants (ROC2-0466, ROC2-0622, ROC2-0870, and both synchronous cancers from patient ROC2-1730), and additional cancers harboring somatic alterations in HR genes such as *BRCA1, BRCA2, WRN, NBN, TONSL, MCM8, and BOD1L1*. Four cancers harbored HR-associated fusion genes (*FUT10::SPIDR, RAD54B::VIRMA, SMARCAD1::BOD1L1*, and *RAD54L::PLXNA2*). In three of these cancers, the fusion co-occurred with other HR-related aberrations, whereas in one cancer (ROC2-1277) the fusion *SMARCAD1::BOD1L1* represented the only detectable HR-associated alteration. Overall, eight of the 15 cancers with HR involvement carried more than one HR-related genomic event, while the remaining cancers showed a single detectable alteration (Table 1).

**Table 1:** Homologous recombination (HR) involvement in high-grade serous tubo-ovarian carcinoma. Integration of pathogenic germline *BRCA1/2* variants, HR deficiency (HRD) signatures, nonsilent somatic mutations, and HR-associated fusion genes (*FUT10::SPIDR, RAD54B::VIRMA, SMARCAD1::BOD1L1,* and *RAD54L::PLXNA2*).

**Patient ID**	**HR involvement**	**HR-related genomic findings**
ROC2-0190	No	Not found
ROC2-0243	Yes	*WRN*, GRCh38:chr8:g.31111801 T > A
ROC2-0308	Yes	*BRCA1*, GRCh38:chr17:g.43063931 G > A; *FUT10::SPIDR*
ROC2-0323	No	Not found
ROC2-0329	Yes	*BRCA2*, GRCh38:chr13:g.32357807 GTTTCACACTG> -; *RAD54B::VIRMA*
ROC2-0379	No	Not found
ROC2-0436	Yes	*NBN*, GRCh38:chr8:g.89971272 A > T
ROC2-0466	Yes	Pathogenic germline variant *BRCA2*; HRD-signature; *TOP3A*, GRCh38:chr17:g.18308882 C > G
ROC2-0479	Yes	HRD-signature
ROC2-0622	Yes	Pathogenic germline variant *BRCA2; HNRNPUL1,* GRCh38:chr19:g.41301662 G > T
ROC2-0766	No	Not found
ROC2-0812	No	Not found
ROC2-0870	Yes	Pathogenic germline variant *BRCA2*; HRD-signature; *PPP4R3A*, GRCh38:chr14:g.91482084 T > C
ROC2-1009	Yes	HRD-signature; *BRCA1*, GRCh38:chr17:g.43047635 CACCTTAC> -; *TONSL*, GRCh38:chr8:g.144442330 C > G
ROC2-1078	Yes	HRD-signature
ROC2-1144	No	Not found
ROC2-1206	No	Not found
ROC2-1277	Yes	*SMARCAD1::BOD1L1*
ROC2-1354	No	Not found
ROC2-1730A	Yes	Pathogenic germline variant *BRCA1*; HRD-signature; *TONSL*, GRCh38:chr8:g.144438479 C > A; *RAD54L::PLXNA2*
ROC2-1730B	Yes	Pathogenic germline variant *BRCA1*; HRD-signature; *MCM8*, GRCh38:chr20:g.5972703 C > T
ROC2-1801	Yes	*BRCA2*, GRCh38:chr13:g.32380043 C > T
ROC2-1914	No	Not found
ROC2-2037	Yes	HRD-signature

## Discussion

The fusion landscape of HGSTOC proved remarkably heterogeneous, with minimal recurrence across patients. The near absence of shared fusion events, with *AKAP8L::BRD4* representing the only example observed in more than one cancer, suggests that fusion formation in this disease is largely determined by patient-specific genomic evolution rather than by common driver mechanisms. The repertoire of partner genes was correspondingly broad, spanning diverse biological functions. While a subset of the fusion transcripts encoded predicted chimeric proteins, the majority were not-in-frame, consistent with previous studies reporting widespread regulatory, rather than protein-coding, consequences of genomic rearrangements in HGSTOC [7,8,32]. This predominance of noncoding and regulatory disruptions aligns with the established biology of HGSTOC, a cancer defined by profound chromosomal instability, extensive structural variation, and near-universal *TP53* mutations [2,33,34]. In our previous study, *TP53* mutations were present in all patients of the current cohort except ROC2-0308 [9].

Virtually all fusion genes were unique to each cancer, in agreement with prior observations that HGSTOC fusion events are largely stochastic consequences of chromosomal instability rather than recurrent oncogenic drivers [7,8]. This scarcity of recurrence limits their value as universal biomarkers or therapeutic targets [35]. Nevertheless, protein kinases were significantly over-represented among fusion partners. Many of these rearrangements preserved intact catalytic domains, indicating that kinase activity may be retained even in the absence of canonical reading-frame continuity. Although many established oncogenic kinase fusions generate in-frame products, out-of-frame rearrangements with biological activity have been documented in *ALK*- and *PDGFRB*-rearranged malignancies [[36], [37], [38], [39]]. More broadly, non-canonical translation mechanisms are increasingly recognized in cancer and developmental contexts, where alternative start codon usage and the production of truncated protein isoforms may contribute to oncogenic signaling diversity [40,41]. The presence of multiple kinase-involving fusions within several cancers therefore suggests repeated perturbation of kinase pathways during cancer evolution. Integration with expression data further demonstrated marked heterogeneity: some cancers with domain-intact kinase fusions belonged to the high-expression group for the respective gene, whereas others showed low expression. Thus, the functional consequences of these events are likely to be strongly context dependent. Even in the absence of recurrence of individual fusions, the repeated involvement of kinases as a functional class highlights potential therapeutic opportunities. Several kinases identified in this cohort, including *BRD4, MTOR, PRKD1, MKNK1*, MAPK4, and *ERBB4*, are targetable with available inhibitors or degraders, suggesting that individualized strategies may be feasible in selected cancers [[42], [43], [44], [45], [46], [47], [48]].

Beyond kinase biology, integrating HRD signatures, germline *BRCA1/2* status, and exome-derived somatic alterations demonstrated that HR involvement was present in nearly two-thirds of the cancers. Many of these harbored multiple alterations, including HRD signatures, pathogenic germline variants, nonsilent HR-gene mutations, and HR-associated fusion genes, suggesting cumulative pathway disruption through parallel genomic events. This pattern emphasizes the benefit of integrating orthogonal molecular information, as reliance on single markers such as *BRCA1/2* would capture only a subset of HR-impaired cancers. Such cancers with HRD signatures or pathogenic *BRCA1/2* variants are established candidates for PARP inhibition, while the contribution of additional HR gene alterations to therapeutic response remains to be clarified.

Taken together, our results indicate that fusion landscapes in HGSTOC are rarely informative at the level of individual recurrent genes. Instead, they reveal recurrent involvement of common pathways. Although specific fusions are largely restricted to single cancers, their collective distribution points to signaling and DNA repair networks important for tumor maintenance and potentially tumor evolution.

This view supports analytical strategies that prioritize functional consequence over recurrence and encourages integrated genomic and transcriptomic interpretation when considering therapeutic opportunities. Determining which alterations represent true vulnerabilities will require systematic experimental and translational validation.

Finally, our study underscores the practical value of multi-algorithm consensus approaches, as requiring support from independent callers proved essential for distinguishing biologically meaningful events from likely artifacts in this highly rearranged tumor type.

## Ethics approval and consent to participate

The project and patient consent for genomic analyses were approved by the Regional Committee for Medical and Health Research Ethics South East Norway (REC no 2014/473). Samples were registered at the Biobank Registry of Norway for tumor tissue (S-04300). All patients provided written informed consent, or exemption from consent was approved for deceased patients.

## Use of artificial intelligence (AI) tools

The AI language model ChatGPT (GPT-5; OpenAI, San Francisco, CA, USA) was used for assistance with Python scripting (data handling and statistical analysis) as well as editorial support for grammar correction and improvement of English readability. The AI tool had no role in study design, data generation, or interpretation of results. All analyses were validated by the author (IP), who takes full responsibility for the content of this manuscript.

## Financial support

The study was supported by grants from The Research Council of Norway (project numbers 250993 and 287899), The Norwegian Cancer Society (project number 308811), and the Southern and Eastern Norway Regional Health Authority (project number 2021082). The study also received grants from Bothners legacy and The Inger and John Fredriksen Foundation for Ovarian Cancer Research (Oslo, Norway). Secure high-performance computational resources were provided by Sigma2 - the National Infrastructure for High Performance Computing and Data Storage, through the allocations NS9013K and NS9013S. The computational work was performed using the Services for Sensitive Data facilities, hosted by the University of Oslo. The funding bodies had no role in the design of the study, nor collection, analysis, or interpretation of data, or in writing the manuscript.

## CRediT authorship contribution statement

**Ioannis Panagopoulos:** Data curation, Formal analysis, Visualization, Writing – original draft, Writing – review & editing. **Ane Stranger:** Data curation, Formal analysis, Visualization, Writing – review & editing. **Anita Sveen:** Formal analysis, Writing – review & editing. **Mie Jareid:** Data curation, Formal analysis, Writing – review & editing. **Susanne G. Kidd:** Data curation, Investigation, Writing – review & editing. **Katharina Bischof:** Writing – review & editing. **Kjetil Taskén:** Writing – review & editing. **Anne Dørum:** Data curation, Resources, Writing – review & editing. **Ben Davidson:** Data curation, Resources, Writing – review & editing. **Ragnhild A. Lothe:** Conceptualization, Methodology, Writing – review & editing. **Rolf I. Skotheim:** Conceptualization, Methodology, Supervision, Writing – review & editing. **Bjarne Johannessen:** Data curation, Formal analysis, Supervision, Writing – review & editing.

## Declaration of competing interest

The authors declare that they have no known competing financial interests or personal relationships that could have appeared to influence the work reported in this paper.

## Data Availability

In accordance with Norwegian legislation and the ethical approval of the study by the Regional Committee for Medical and Health Research Ethics South East Norway, the raw high-throughput sequencing data generated in this study are considered patient identifiable and subject to secure storage regulations in accordance with the national Personal Data Regulations, chapter 2. Data can currently not be deposited to public repositories. Data will be made available upon reasonable request to BJ, and this will require formalization of a data transfer agreement. All analyses were performed with published software packages and computer code and are described in the Methods.

## Appendix Supplementary materials

Image, application 1

Image, application 2
