# Supplementary material for: Fusion gene heterogeneity and kinase enrichment in high-grade serous carcinomas
Source: Neoplasia. 2026 Jun 25;79:101332. doi: 10.1016/j.neo.2026.101332 (PMC13325326; doi:10.1016/j.neo.2026.101332)
Supplement: Supplementary file 1 [file mmc1.pdf]

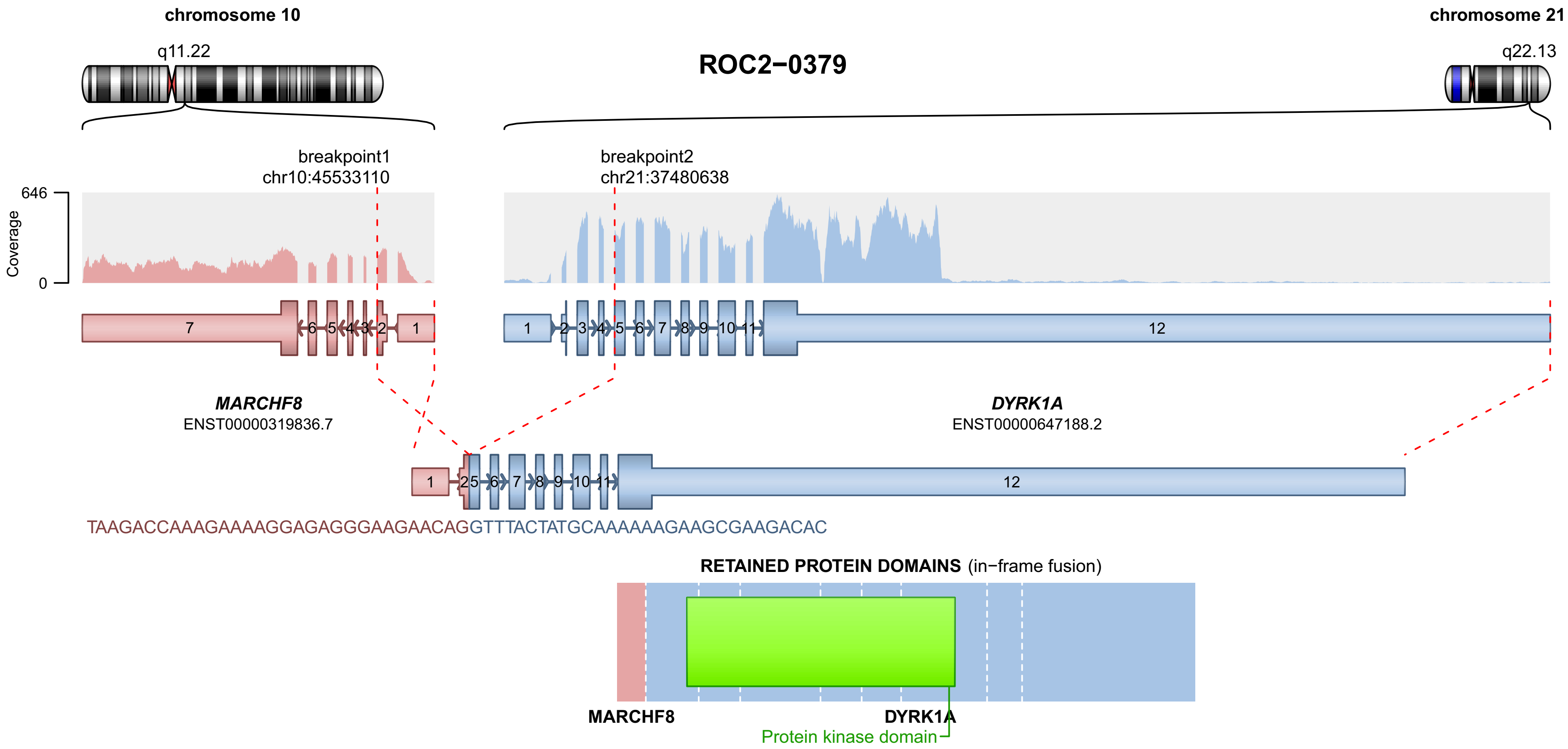

Supplementary Figure S1. The detected *MARCHF8::DYRK1A* fusion in patient ROC2-0379.

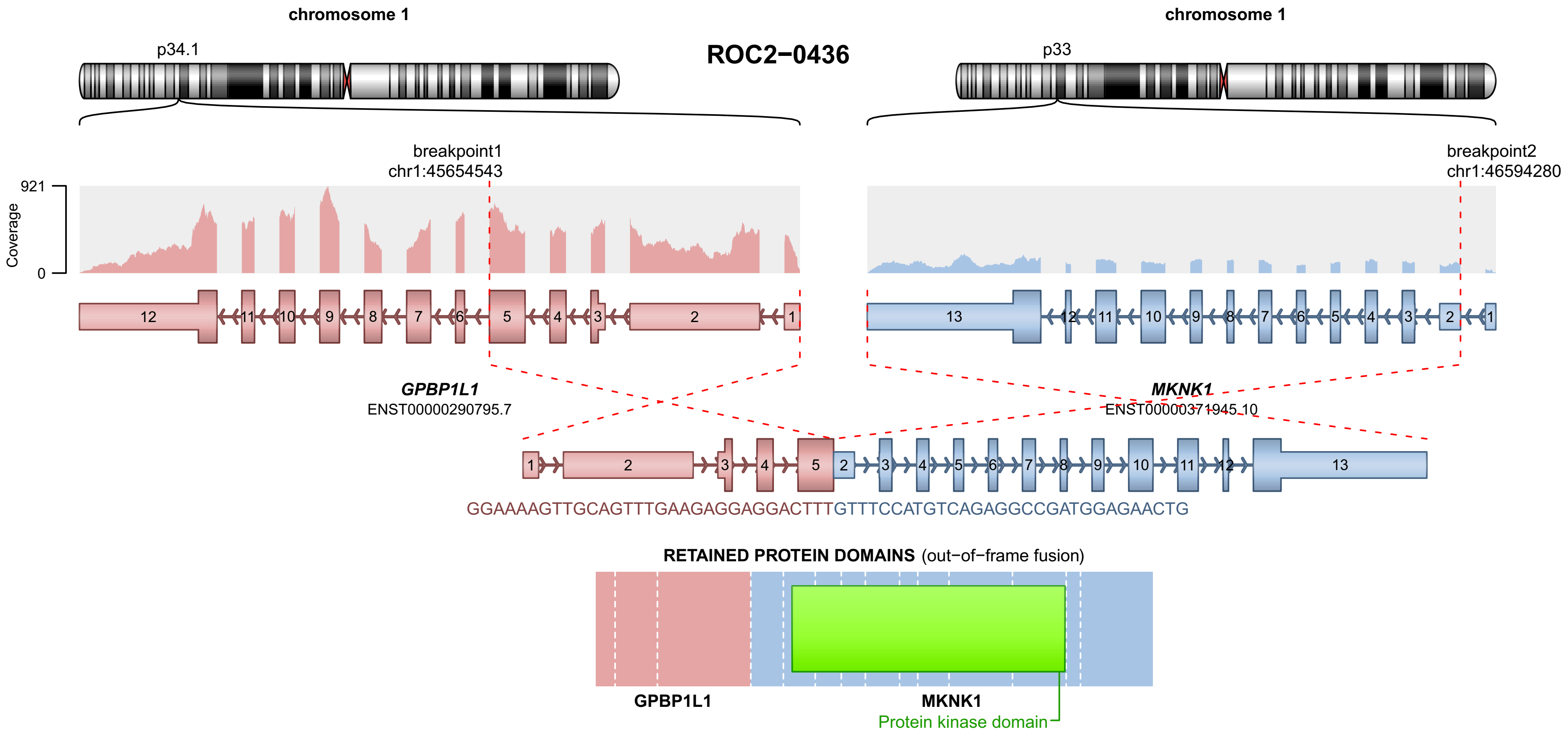

Supplementary Figure S2. The detected *GPBP1L1::MKNK1* fusion in patient ROC2-0436.

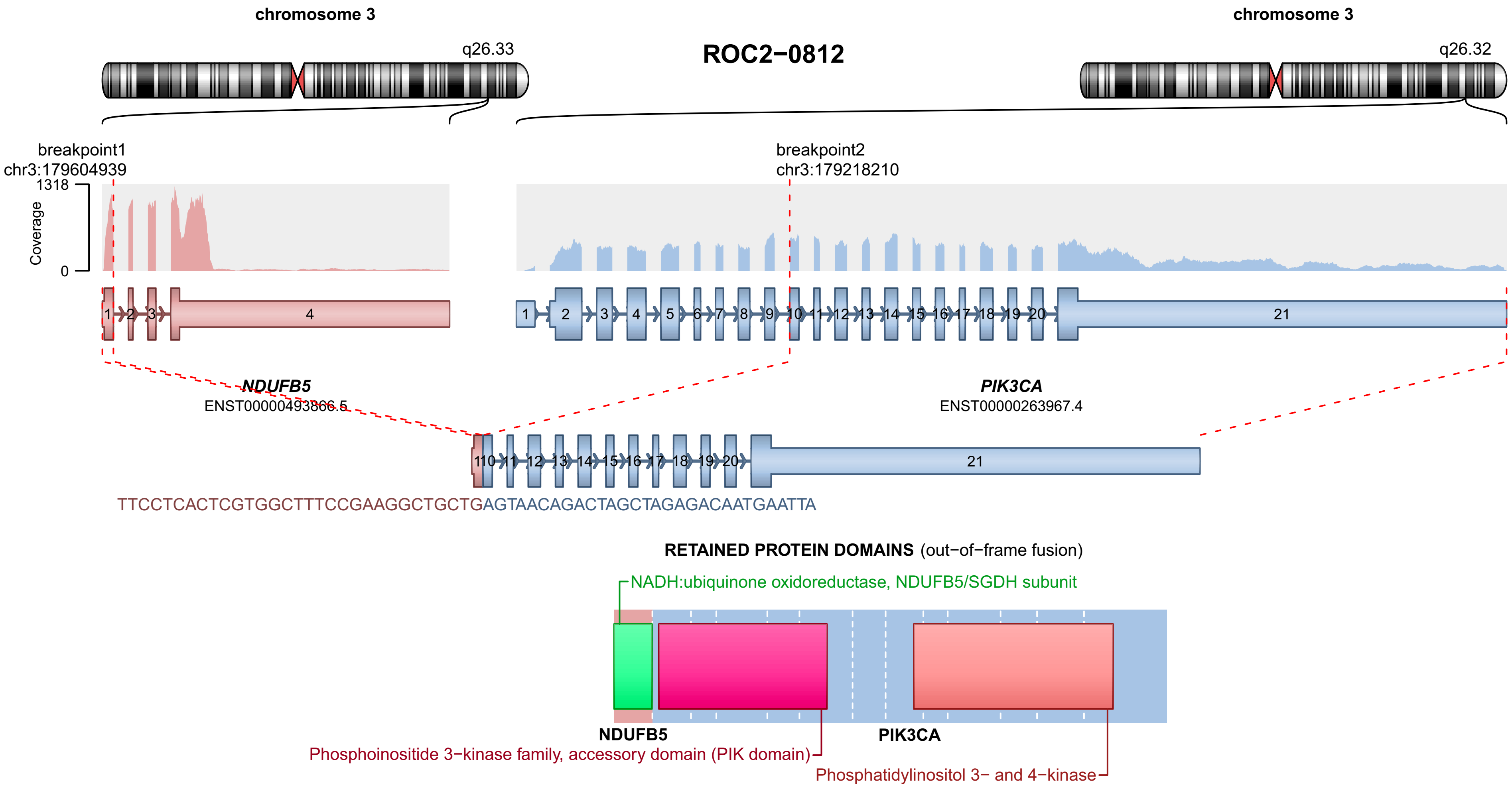

Supplementary Figure S3. The detected *NDUFB5::PIK3CA* fusion in patient ROC2-0812.

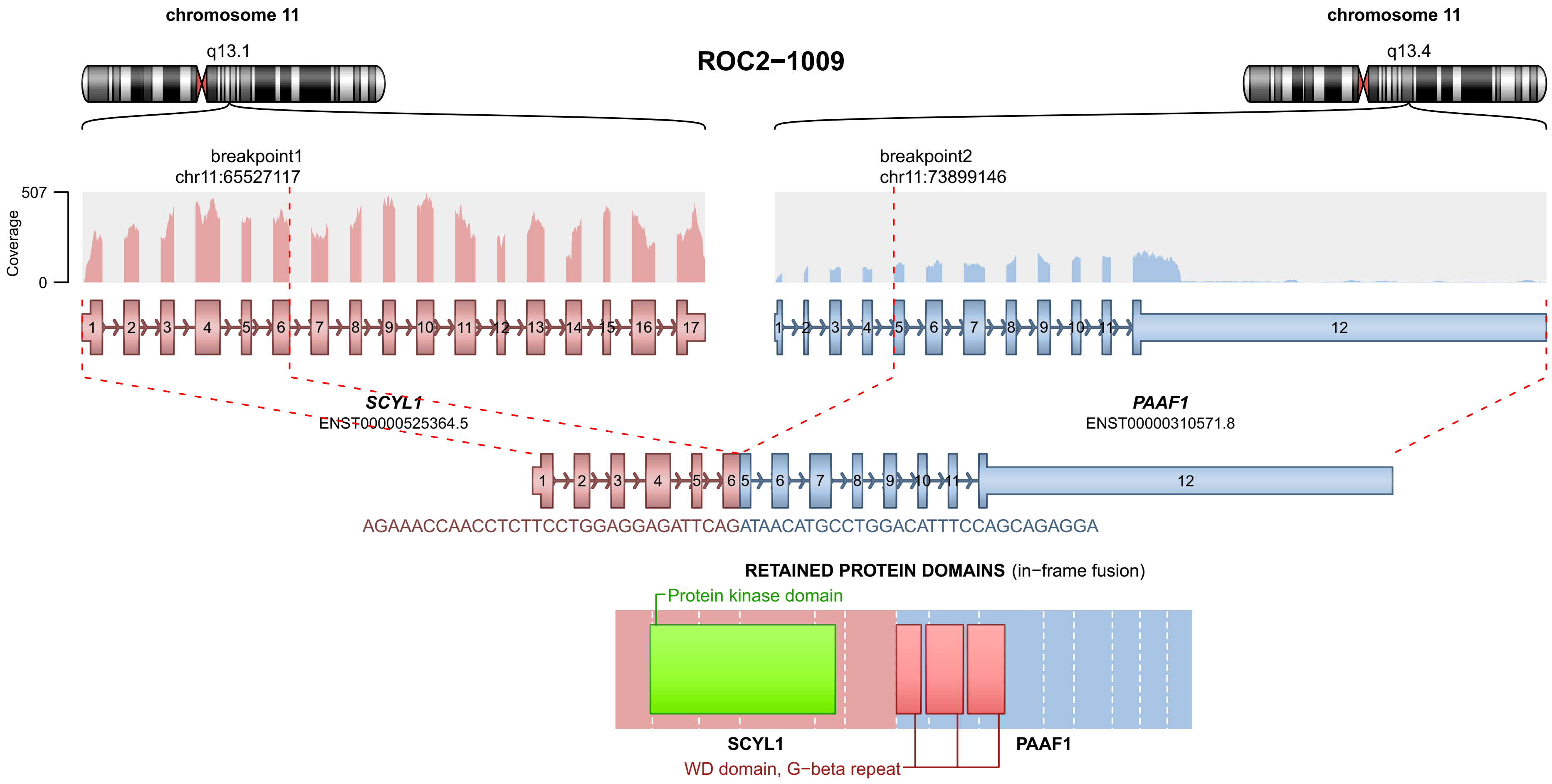

Supplementary Figure S4. The detected *SCYL1::PAAF1* fusion in patient ROC2-1009.

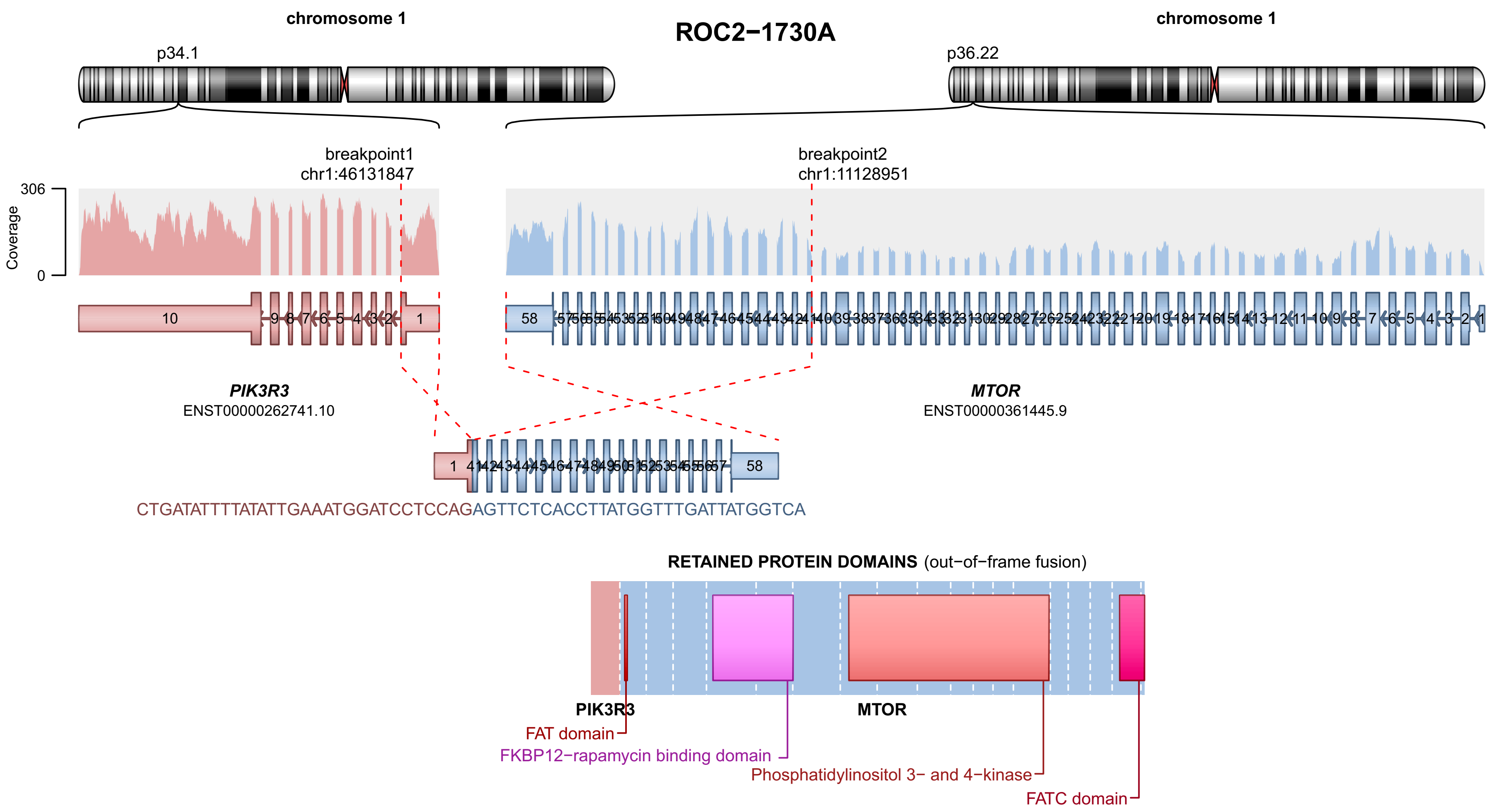

Supplementary Figure S5. The detected *PIK3R3::MTOR* fusion in cancer ROC2-1730A (patient ROC2-1730).

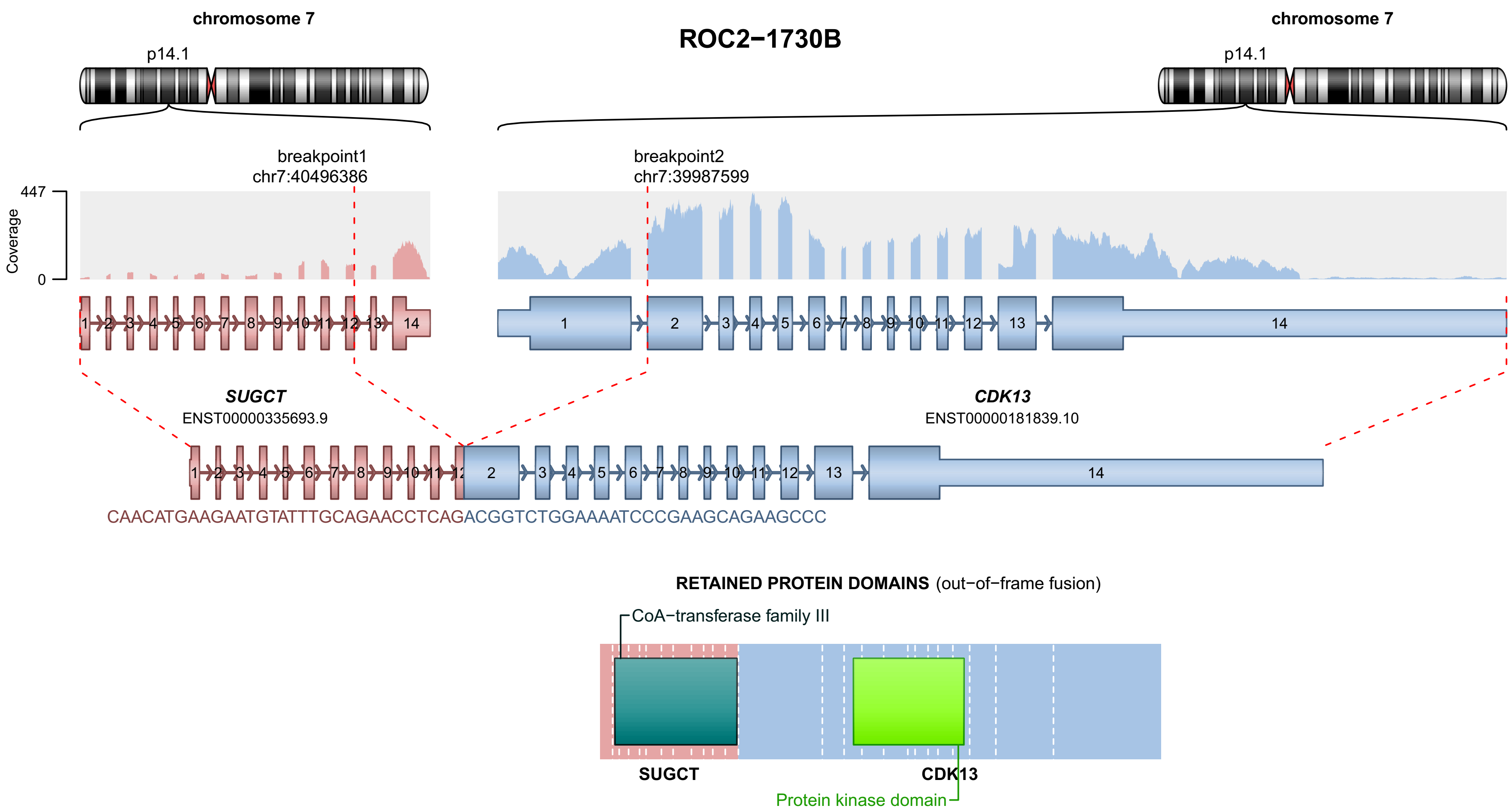

Supplementary Figure S6. The detected *SUGCT::CDK13* fusion in cancer ROC2-1730B (patient ROC2-1730).

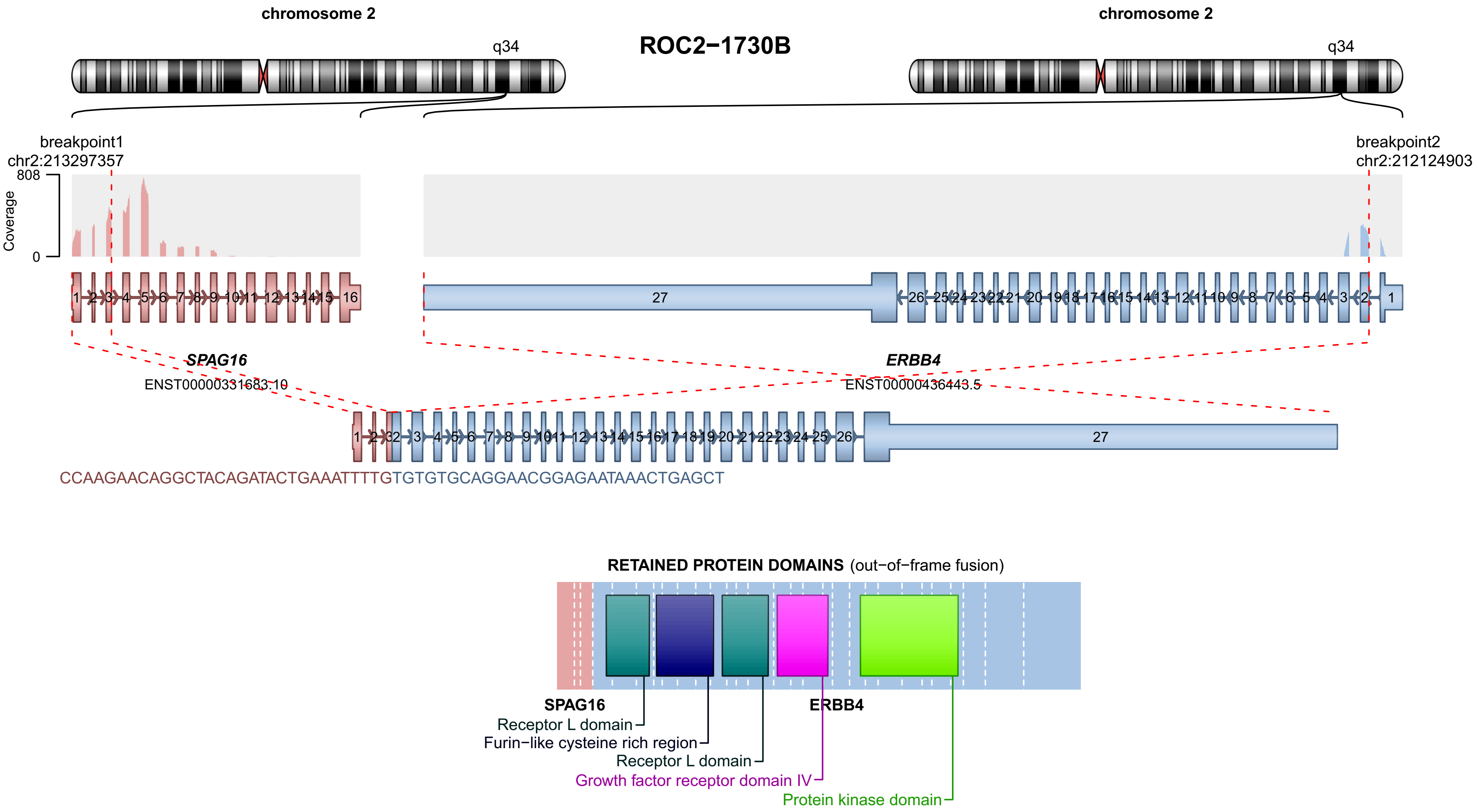

Supplementary Figure S7. The detected *SPAG16::ERBB4* fusion in cancer ROC2-1730B (patient ROC2-1730).

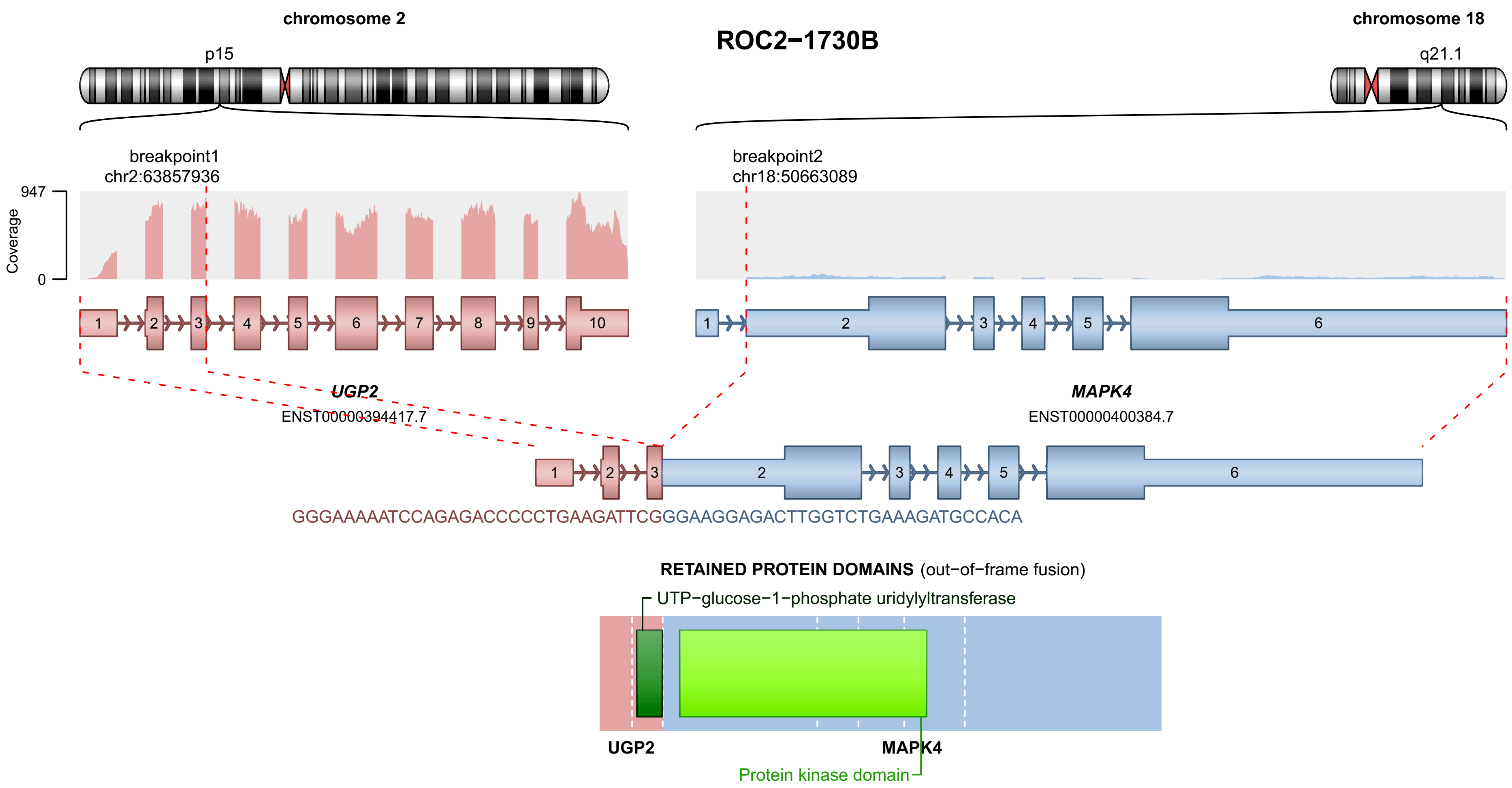

Supplementary Figure S8. The detected *UGP2::MAPK4* fusion in cancer ROC2-1730B (patient ROC2-1730).

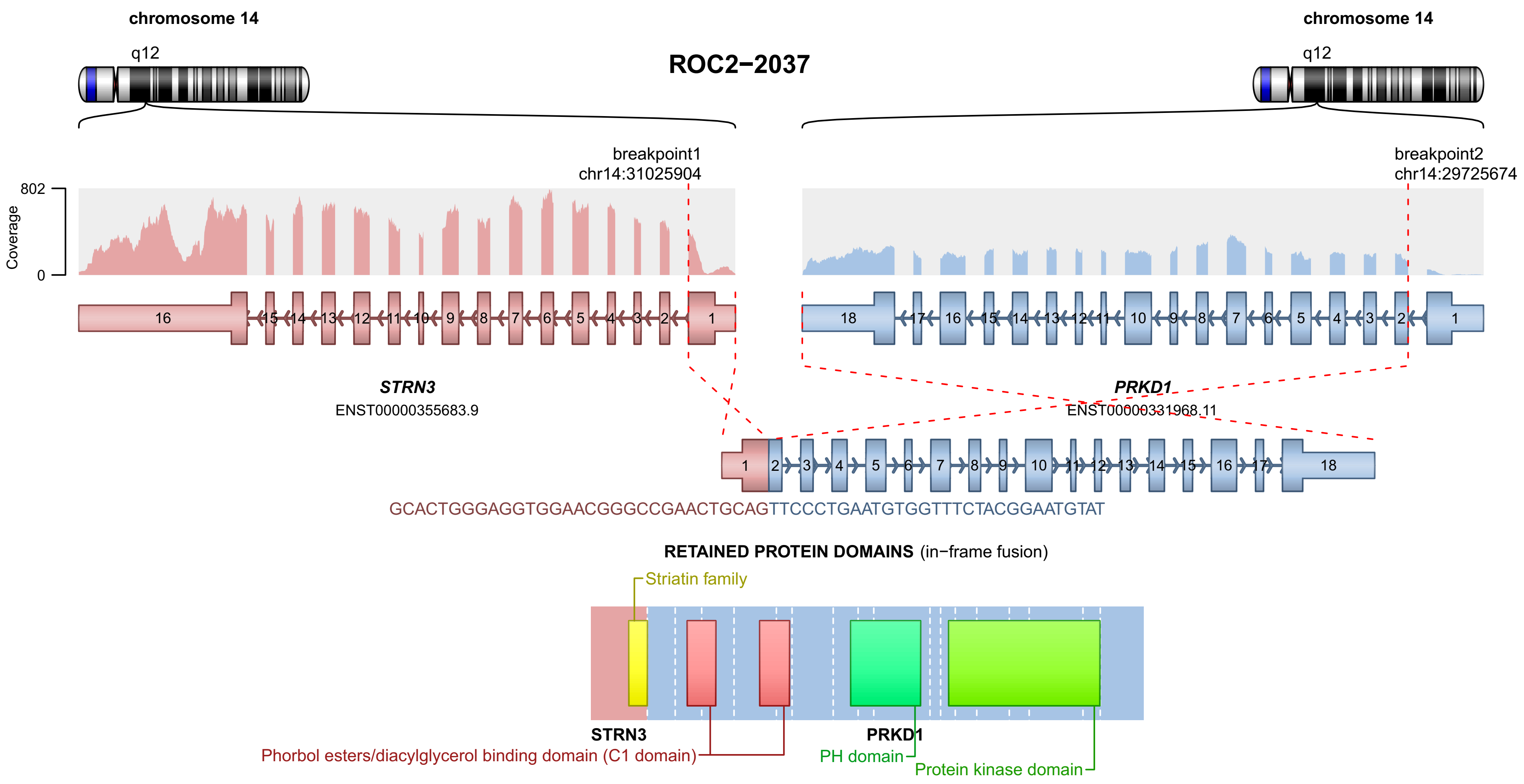

Supplementary Figure S9. The detected *STRN3::PRKD1* fusion in patient ROC2-2037.
